# Poly[(5,5′-dimethyl-2,2′-bipyridine-κ^2^ *N*,*N*′)(μ_3_-5-hy­droxy­isophthalato-κ^4^ *O* ^1^:*O* ^3^,*O* ^3′^:*O* ^3′^)cadmium]

**DOI:** 10.1107/S1600536811035975

**Published:** 2011-09-14

**Authors:** Xi-Ying Hu, Wen-Hang Zhai, Ning Ma, Guang-Rui Yang

**Affiliations:** a North China University of Water Conservancy and Electric Power, Zhengzhou 450011, People’s Republic of China

## Abstract

In the title compound, [Cd(C_8_H_4_O_5_)(C_12_H_12_N_2_)], the Cd^II^ cation is coordinated by three 5-hy­droxy­isophthalate anions and one 5,5′-bimethyl-2,2′-bipyridine ligand in a distorted CdO_4_N_2_ octa­hedral geometry. The 5-hy­droxy­isophthalate anions bridge the Cd cations, forming a two-dimensional polymeric complex parallel to (100). In the complex, the hy­droxy group is linked to the uncoordinated carb­oxy-O atom *via* an O—H⋯O hydrogen bond. Weak C—H⋯O hydrogen bonds are also present in the crystal structure. One of the methyl groups is disordered over two positions in a 0.536 (11):0.464 (11) ratio.

## Related literature

For background to network topologies and applications of coordination polymers, see: Maspoch *et al.* (2007 ▶); Ockwig *et al.* (2005 ▶); Zang *et al.* (2011 ▶).
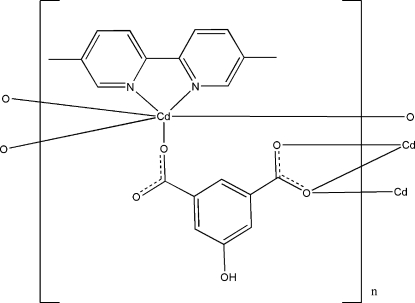


## Experimental

### 

#### Crystal data

[Cd(C_8_H_4_O_5_)(C_12_H_12_N_2_)]
*M* *_r_* = 476.75
Monoclinic, 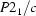

*a* = 10.7650 (2) Å
*b* = 13.0111 (3) Å
*c* = 16.5272 (4) Å
β = 125.235 (2)°
*V* = 1890.77 (7) Å^3^
*Z* = 4
Mo *K*α radiation
μ = 1.19 mm^−1^
*T* = 296 K
0.21 × 0.20 × 0.19 mm

#### Data collection

Bruker APEXII CCD area detector diffractometer
Absorption correction: multi-scan (*SADABS*; Bruker, 2005 ▶) *T*_min_ = 0.788, *T*_max_ = 0.806
7327 measured reflections
3315 independent reflections
2963 reflections with *I* > 2σ(*I*)
*R*_int_ = 0.022

#### Refinement

*R*[*F* ^2^ > 2σ(*F* ^2^)] = 0.024
*wR*(*F* ^2^) = 0.063
*S* = 1.03
3315 reflections
261 parameters
19 restraints
H-atom parameters constrained
Δρ_max_ = 0.44 e Å^−3^
Δρ_min_ = −0.49 e Å^−3^

### 

Data collection: *APEX2* (Bruker, 2005 ▶); cell refinement: *SAINT* (Bruker, 2005 ▶); data reduction: *SAINT*; program(s) used to solve structure: *SHELXTL* (Sheldrick, 2008 ▶); program(s) used to refine structure: *SHELXTL*; molecular graphics: *SHELXTL*; software used to prepare material for publication: *SHELXTL*.

## Supplementary Material

Crystal structure: contains datablock(s) I, global. DOI: 10.1107/S1600536811035975/xu5315sup1.cif

Structure factors: contains datablock(s) I. DOI: 10.1107/S1600536811035975/xu5315Isup2.hkl

Additional supplementary materials:  crystallographic information; 3D view; checkCIF report

## Figures and Tables

**Table 1:** Selected bond lengths (Å)

Cd1—O1	2.1884 (19)
Cd1—O3^i^	2.4015 (19)
Cd1—O4^i^	2.3209 (18)
Cd1—O4^ii^	2.3922 (19)
Cd1—N1	2.329 (2)
Cd1—N2	2.340 (2)

Symmetry codes: (i) 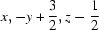; (ii) 
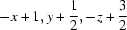
.

**Table 2:** Hydrogen-bond geometry (Å, °)

*D*—H⋯*A*	*D*—H	H⋯*A*	*D*⋯*A*	*D*—H⋯*A*
O5—H5⋯O2^iii^	0.82	1.86	2.680 (3)	174
C6—H6⋯O1^iv^	0.93	2.31	3.229 (3)	169
C17—H17⋯O3^v^	0.93	2.53	3.355 (5)	147

Symmetry codes: (iii) 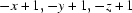; (iv) 
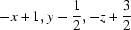
; (v) 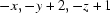.

**Fig. 1.**
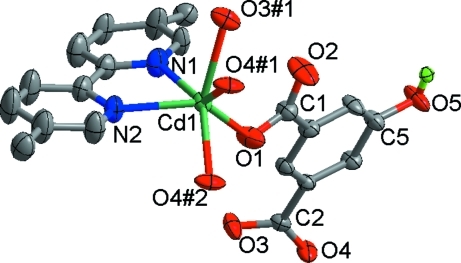
Metal coordination and atom labeling in title compound (thermal ellipsoids at 50% probability level). Irrespective hydrogen atoms are omitted for clarity.

**Fig. 2.**
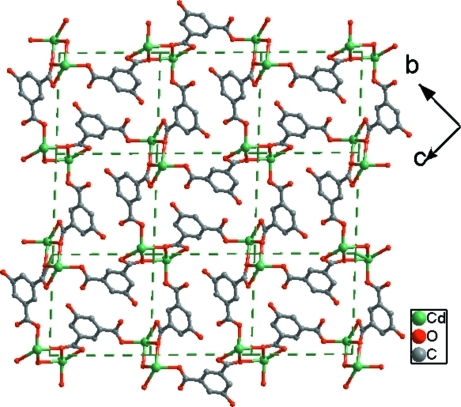
A view of the layer in compound 1. Dotted lines represent the topological view of the layer structure. The bmbpy ligands are omitted for clarity.

## supplementary crystallographic information

### Comment

In recent years, supramolecular coordination assemblies have received much attention due to their variety of architectures and the potential applications as functional materials (Maspoch *et al.*, 2007; Ockwig *et al.*, 2005). A great number of isophthalic acid and its derivatives have been successfully employed in the generation of many novel structures (Zang *et al.*, 2011). To further explore various factors that influence the properties and construction of coordination compounds, we undertake synthetic and structural studies on one novel Cd(II) complex based on 5-hydroxyisophthalic acid (H_2_hip) and 5,5'-bimethyl-2,2'-bipyridine(bmbpy): Cd(hip)(bmbpy) (1).

As shown in Fig. 1, the asymmetric unit consists of one Cd^II^ atom, one hip^2-^ anion and one dmbpy ligand. The Cd^II^ atom is six-coordinated by four O atoms from three 5-hydroxyisophthalate ligands and two N atoms from a chelating 5,5'-bimethyl-2,2'-bipyridine ligand. Each hip^2-^ ligand acts as a *µ*_3_-bridge linking three Cd^II^ atoms with one carboxylate groups in monodentate fashion and the other one in chelating/bridging mode. As depicted in Fig. 2, pair of metal atoms are linked together through two carboxylate oxygen atoms to form a tetratomic ring Cd_2_O_2_. Adjacent rings are further connected by hip^2-^ ligands to result in a layer structure in *bc* plane with the N-donor ligands hanging from it. A better understanding of this structure can be achieved *via* topological considerations. If the hip^2-^ ligand are considered as connecters, and the Cd_2_O_2_ Units are considered as four-connected nodes (connecting to four other such units *via* hip^2-^ ligands), the layer structure of 1 can be described as a (4,4)-net.

### Experimental

Compound 1 was synthesized hydrothermally in a Teflon-lined stainless steel container by heating a mixture of 5-hydroxyisophthalic acid (H_2_hip) (0.0091 g, 0.05 mmol), 5,5'-bimethyl-2,2'-bipyridine(bmbpy) (0.0092 g, 0.05 mmol), Cd(NO_3_)_2_.4H_2_O (0.0154 g, 0.05 mmol) and NaOH (0.0040 g, 0.1 mmol) in 7 ml of distilled water at 120°C for 3 days, and then cooled to room temperature. Colorless block crystals of 1 were obtained in 69% yield based on cadmium.

### Refinement

H atoms were positioned geometrically and refined using a riding model, with C—H = 0.93–0.96 Å, *U*_iso_(H) = 1.2*U*_eq_(C) for aromatic H atoms and 1.5*U*_eq_(C,O) for methyl and hydroxy H atoms.

### Crystal data

**Table:**

[Cd(C_8_H_4_O_5_)(C_12_H_12_N_2_)]	*F*(000) = 952
*M**_r_* = 476.75	*D*_x_ = 1.675 Mg m^−^^3^
Monoclinic, *P*2_1_/*c*	Mo *K*α radiation, λ = 0.71073 Å
Hall symbol: -P 2ybc	Cell parameters from 4955 reflections
*a* = 10.7650 (2) Å	θ = 3.0–29.2°
*b* = 13.0111 (3) Å	µ = 1.19 mm^−^^1^
*c* = 16.5272 (4) Å	*T* = 296 K
β = 125.235 (2)°	Block, colourless
*V* = 1890.77 (7) Å^3^	0.21 × 0.20 × 0.19 mm
*Z* = 4	

### Data collection

**Table:**

Bruker APEXII CCD area detector diffractometer	3315 independent reflections
Radiation source: fine-focus sealed tube	2963 reflections with *I* > 2σ(*I*)
graphite	*R*_int_ = 0.022
ω scans	θ_max_ = 25.0°, θ_min_ = 3.0°
Absorption correction: multi-scan (*SADABS*; Bruker, 2005)	*h* = −12→12
*T*_min_ = 0.788, *T*_max_ = 0.806	*k* = −13→15
7327 measured reflections	*l* = −18→19

### Refinement

**Table:**

Refinement on *F*^2^	Primary atom site location: structure-invariant direct methods
Least-squares matrix: full	Secondary atom site location: difference Fourier map
*R*[*F*^2^ > 2σ(*F*^2^)] = 0.024	Hydrogen site location: inferred from neighbouring sites
*wR*(*F*^2^) = 0.063	H-atom parameters constrained
*S* = 1.03	*w* = 1/[σ^2^(*F*_o_^2^) + (0.0329*P*)^2^ + 0.440*P*] where *P* = (*F*_o_^2^ + 2*F*_c_^2^)/3
3315 reflections	(Δ/σ)_max_ = 0.002
261 parameters	Δρ_max_ = 0.44 e Å^−^^3^
19 restraints	Δρ_min_ = −0.49 e Å^−^^3^

### Special details

**Table:**

Geometry. All e.s.d.'s (except the e.s.d. in the dihedral angle between two l.s. planes) are estimated using the full covariance matrix. The cell e.s.d.'s are taken into account individually in the estimation of e.s.d.'s in distances, angles and torsion angles; correlations between e.s.d.'s in cell parameters are only used when they are defined by crystal symmetry. An approximate (isotropic) treatment of cell e.s.d.'s is used for estimating e.s.d.'s involving l.s. planes.
Refinement. Refinement of *F*^2^ against ALL reflections. The weighted *R*-factor *wR* and goodness of fit *S* are based on *F*^2^, conventional *R*-factors *R* are based on *F*, with *F* set to zero for negative *F*^2^. The threshold expression of *F*^2^ > σ(*F*^2^) is used only for calculating *R*-factors(gt) *etc*. and is not relevant to the choice of reflections for refinement. *R*-factors based on *F*^2^ are statistically about twice as large as those based on *F*, and *R*- factors based on ALL data will be even larger.

### Fractional atomic coordinates and isotropic or equivalent isotropic displacement parameters (Å^2^)

**Table:**

	*x*	*y*	*z*	*U*_iso_*/*U*_eq_	Occ. (<1)
Cd1	0.33624 (2)	0.908899 (14)	0.465227 (14)	0.02455 (9)	
O1	0.4236 (3)	0.79166 (15)	0.58085 (15)	0.0445 (5)	
O2	0.4437 (3)	0.68188 (19)	0.48809 (18)	0.0605 (7)	
O3	0.3021 (2)	0.63401 (16)	0.81224 (15)	0.0398 (5)	
O4	0.4714 (2)	0.51992 (16)	0.90921 (14)	0.0380 (5)	
O5	0.5449 (3)	0.34087 (16)	0.66869 (17)	0.0482 (6)	
H5	0.5532	0.3368	0.6225	0.072*	
N1	0.0946 (3)	0.8445 (2)	0.4020 (2)	0.0447 (7)	
N2	0.1680 (3)	1.0450 (2)	0.42515 (19)	0.0387 (6)	
C1	0.4423 (3)	0.7026 (2)	0.5600 (2)	0.0311 (6)	
C2	0.4028 (3)	0.5714 (2)	0.83066 (19)	0.0234 (6)	
C3	0.4561 (3)	0.6172 (2)	0.62673 (19)	0.0268 (6)	
C4	0.4971 (3)	0.5188 (2)	0.6169 (2)	0.0331 (7)	
H4	0.5166	0.5067	0.5697	0.040*	
C5	0.5091 (3)	0.4393 (2)	0.6761 (2)	0.0310 (6)	
C6	0.4842 (3)	0.4582 (2)	0.74822 (19)	0.0269 (6)	
H6	0.4975	0.4059	0.7909	0.032*	
C7	0.4394 (3)	0.5551 (2)	0.75669 (18)	0.0224 (5)	
C8	0.4245 (3)	0.6349 (2)	0.69598 (19)	0.0251 (6)	
H8	0.3936	0.6997	0.7016	0.030*	
C9	0.0650 (4)	0.7438 (3)	0.3945 (3)	0.0666 (12)	
H9	0.1411	0.6988	0.4064	0.080*	
C10	−0.0689 (4)	0.7022 (3)	0.3704 (4)	0.0818 (15)	
C11	−0.0818 (12)	0.5869 (7)	0.3935 (10)	0.0662 (17)	0.464 (11)
H11A	−0.0717	0.5426	0.3511	0.099*	0.464 (11)
H11B	−0.1789	0.5758	0.3820	0.099*	0.464 (11)
H11C	−0.0024	0.5720	0.4614	0.099*	0.464 (11)
C11'	−0.0983 (10)	0.5886 (6)	0.3397 (9)	0.0662 (17)	0.536 (11)
H11D	−0.1467	0.5832	0.2697	0.099*	0.536 (11)
H11E	−0.1632	0.5594	0.3560	0.099*	0.536 (11)
H11F	−0.0034	0.5521	0.3744	0.099*	0.536 (11)
C12	−0.1789 (4)	0.7708 (3)	0.3531 (3)	0.0741 (13)	
H12	−0.2718	0.7465	0.3372	0.089*	
C13	−0.1532 (4)	0.8742 (3)	0.3590 (3)	0.0603 (11)	
H13	−0.2283	0.9202	0.3470	0.072*	
C14	−0.0139 (3)	0.9107 (2)	0.3832 (2)	0.0395 (8)	
C15	0.0231 (3)	1.0213 (3)	0.3885 (2)	0.0399 (7)	
C16	−0.0843 (4)	1.0982 (3)	0.3547 (3)	0.0659 (12)	
H16	−0.1840	1.0817	0.3308	0.079*	
C17	−0.0446 (4)	1.1988 (3)	0.3560 (3)	0.0726 (13)	
H17	−0.1181	1.2500	0.3316	0.087*	
C18	0.1026 (4)	1.2240 (3)	0.3932 (3)	0.0629 (11)	
C19	0.1526 (5)	1.3337 (3)	0.3959 (4)	0.0828 (13)	
H19A	0.0711	1.3708	0.3399	0.124*	
H19B	0.2396	1.3334	0.3935	0.124*	
H19C	0.1787	1.3663	0.4559	0.124*	
C20	0.2049 (4)	1.1434 (3)	0.4275 (3)	0.0523 (9)	
H20	0.3059	1.1587	0.4539	0.063*	

### Atomic displacement parameters (Å^2^)

**Table:**

	*U*^11^	*U*^22^	*U*^33^	*U*^12^	*U*^13^	*U*^23^
Cd1	0.03011 (13)	0.02303 (13)	0.02889 (13)	0.00030 (8)	0.02186 (10)	0.00374 (8)
O1	0.0808 (15)	0.0212 (11)	0.0346 (12)	0.0003 (10)	0.0351 (11)	0.0044 (9)
O2	0.115 (2)	0.0487 (15)	0.0543 (15)	0.0187 (14)	0.0701 (16)	0.0163 (12)
O3	0.0564 (13)	0.0369 (12)	0.0468 (13)	0.0183 (10)	0.0417 (11)	0.0115 (10)
O4	0.0502 (12)	0.0473 (13)	0.0265 (10)	0.0206 (10)	0.0279 (9)	0.0125 (10)
O5	0.0970 (17)	0.0237 (11)	0.0525 (14)	0.0176 (11)	0.0596 (14)	0.0079 (10)
N1	0.0337 (13)	0.0348 (15)	0.0647 (18)	−0.0030 (12)	0.0279 (13)	−0.0001 (14)
N2	0.0351 (13)	0.0308 (14)	0.0513 (16)	0.0056 (11)	0.0256 (12)	0.0057 (13)
C1	0.0402 (15)	0.0273 (16)	0.0303 (15)	0.0009 (12)	0.0228 (13)	0.0032 (13)
C2	0.0282 (13)	0.0213 (14)	0.0248 (14)	−0.0052 (11)	0.0177 (12)	−0.0029 (12)
C3	0.0359 (14)	0.0242 (14)	0.0236 (14)	−0.0002 (12)	0.0190 (12)	0.0036 (12)
C4	0.0559 (18)	0.0275 (15)	0.0313 (15)	0.0052 (13)	0.0340 (14)	0.0012 (13)
C5	0.0469 (17)	0.0227 (14)	0.0334 (16)	0.0050 (12)	0.0290 (14)	0.0008 (13)
C6	0.0375 (15)	0.0238 (15)	0.0251 (14)	0.0015 (12)	0.0214 (12)	0.0055 (12)
C7	0.0256 (13)	0.0228 (13)	0.0214 (13)	−0.0001 (11)	0.0150 (11)	0.0001 (11)
C8	0.0328 (14)	0.0183 (13)	0.0268 (14)	0.0019 (11)	0.0187 (12)	−0.0003 (12)
C9	0.0417 (19)	0.041 (2)	0.107 (3)	−0.0020 (16)	0.037 (2)	−0.005 (2)
C10	0.042 (2)	0.044 (2)	0.130 (4)	−0.0106 (18)	0.033 (2)	0.003 (3)
C11	0.0641 (19)	0.061 (2)	0.069 (2)	−0.0056 (12)	0.0358 (15)	0.0007 (15)
C11'	0.0641 (19)	0.061 (2)	0.069 (2)	−0.0056 (12)	0.0358 (15)	0.0007 (15)
C12	0.0377 (19)	0.059 (3)	0.113 (4)	−0.0105 (18)	0.037 (2)	0.007 (3)
C13	0.0345 (18)	0.060 (2)	0.083 (3)	0.0047 (17)	0.0316 (19)	0.006 (2)
C14	0.0296 (15)	0.0428 (19)	0.0448 (19)	0.0010 (13)	0.0208 (14)	0.0018 (15)
C15	0.0307 (15)	0.0424 (19)	0.0438 (18)	0.0052 (14)	0.0199 (14)	0.0050 (15)
C16	0.0374 (19)	0.050 (2)	0.093 (3)	0.0121 (16)	0.028 (2)	0.009 (2)
C17	0.050 (2)	0.052 (3)	0.097 (3)	0.0230 (19)	0.032 (2)	0.013 (2)
C18	0.053 (2)	0.0414 (18)	0.081 (3)	0.0153 (16)	0.031 (2)	0.011 (2)
C19	0.0794 (19)	0.0575 (17)	0.096 (2)	0.0031 (14)	0.0418 (15)	0.0074 (16)
C20	0.0410 (18)	0.0338 (19)	0.076 (3)	0.0032 (15)	0.0304 (18)	0.0059 (19)

### Geometric parameters (Å, °)

**Table:**

Cd1—O1	2.1884 (19)	C7—C8	1.387 (4)
Cd1—O3^i^	2.4015 (19)	C8—H8	0.9300
Cd1—O4^i^	2.3209 (18)	C9—C10	1.367 (5)
Cd1—O4^ii^	2.3922 (19)	C9—H9	0.9300
Cd1—N1	2.329 (2)	C10—C12	1.374 (6)
Cd1—N2	2.340 (2)	C10—C11'	1.536 (9)
O1—C1	1.257 (3)	C10—C11	1.574 (10)
O2—C1	1.229 (3)	C11—H11A	0.9600
O3—C2	1.246 (3)	C11—H11B	0.9600
O3—Cd1^iii^	2.4015 (19)	C11—H11C	0.9600
O4—C2	1.254 (3)	C11'—H11D	0.9600
O4—Cd1^iii^	2.3209 (18)	C11'—H11E	0.9600
O4—Cd1^iv^	2.3922 (18)	C11'—H11F	0.9600
O5—C5	1.363 (3)	C12—C13	1.367 (6)
O5—H5	0.8200	C12—H12	0.9300
N1—C9	1.337 (4)	C13—C14	1.394 (5)
N1—C14	1.335 (4)	C13—H13	0.9300
N2—C20	1.333 (4)	C14—C15	1.482 (4)
N2—C15	1.343 (4)	C15—C16	1.380 (5)
C1—C3	1.511 (4)	C16—C17	1.373 (5)
C2—C7	1.500 (3)	C16—H16	0.9300
C2—Cd1^iii^	2.714 (3)	C17—C18	1.368 (5)
C3—C8	1.389 (4)	C17—H17	0.9300
C3—C4	1.394 (4)	C18—C20	1.384 (5)
C4—C5	1.379 (4)	C18—C19	1.517 (6)
C4—H4	0.9300	C19—H19A	0.9600
C5—C6	1.386 (4)	C19—H19B	0.9600
C6—C7	1.386 (4)	C19—H19C	0.9600
C6—H6	0.9300	C20—H20	0.9300
			
O1—Cd1—O4^i^	125.02 (8)	C6—C7—C8	120.6 (2)
O1—Cd1—N1	86.97 (9)	C6—C7—C2	118.8 (2)
O4^i^—Cd1—N1	139.44 (9)	C8—C7—C2	120.6 (2)
O1—Cd1—N2	130.36 (9)	C7—C8—C3	119.4 (2)
O4^i^—Cd1—N2	98.40 (8)	C7—C8—H8	120.3
N1—Cd1—N2	70.33 (9)	C3—C8—H8	120.3
O1—Cd1—O4^ii^	86.70 (7)	N1—C9—C10	124.7 (3)
O4^i^—Cd1—O4^ii^	71.16 (7)	N1—C9—H9	117.7
N1—Cd1—O4^ii^	142.19 (8)	C10—C9—H9	117.7
N2—Cd1—O4^ii^	85.97 (8)	C9—C10—C12	116.2 (4)
O1—Cd1—O3^i^	119.01 (8)	C9—C10—C11'	117.3 (5)
O4^i^—Cd1—O3^i^	54.77 (6)	C12—C10—C11'	124.6 (4)
N1—Cd1—O3^i^	89.50 (8)	C9—C10—C11	122.1 (5)
N2—Cd1—O3^i^	104.75 (8)	C12—C10—C11	118.9 (5)
O4^ii^—Cd1—O3^i^	125.72 (6)	C10—C11—H11A	109.5
O1—Cd1—C2^i^	126.71 (8)	C10—C11—H11B	109.5
O4^i^—Cd1—C2^i^	27.45 (7)	C10—C11—H11C	109.5
N1—Cd1—C2^i^	114.91 (9)	C10—C11'—H11D	109.5
N2—Cd1—C2^i^	102.94 (8)	C10—C11'—H11E	109.5
O4^ii^—Cd1—C2^i^	98.53 (7)	H11D—C11'—H11E	109.5
O3^i^—Cd1—C2^i^	27.32 (7)	C10—C11'—H11F	109.5
C1—O1—Cd1	117.35 (18)	H11D—C11'—H11F	109.5
C2—O3—Cd1^iii^	90.45 (16)	H11E—C11'—H11F	109.5
C2—O4—Cd1^iii^	93.99 (15)	C13—C12—C10	120.6 (3)
C2—O4—Cd1^iv^	156.69 (17)	C13—C12—H12	119.7
Cd1^iii^—O4—Cd1^iv^	108.84 (7)	C10—C12—H12	119.7
C5—O5—H5	109.5	C12—C13—C14	119.7 (3)
C9—N1—C14	118.8 (3)	C12—C13—H13	120.1
C9—N1—Cd1	122.6 (2)	C14—C13—H13	120.1
C14—N1—Cd1	118.2 (2)	N1—C14—C13	119.9 (3)
C20—N2—C15	118.9 (3)	N1—C14—C15	116.3 (3)
C20—N2—Cd1	123.3 (2)	C13—C14—C15	123.7 (3)
C15—N2—Cd1	117.5 (2)	N2—C15—C16	120.0 (3)
O2—C1—O1	124.1 (3)	N2—C15—C14	116.9 (3)
O2—C1—C3	119.6 (3)	C16—C15—C14	123.1 (3)
O1—C1—C3	116.3 (2)	C17—C16—C15	120.3 (3)
O3—C2—O4	120.8 (2)	C17—C16—H16	119.9
O3—C2—C7	119.6 (2)	C15—C16—H16	119.9
O4—C2—C7	119.6 (2)	C18—C17—C16	120.3 (3)
O3—C2—Cd1^iii^	62.23 (14)	C18—C17—H17	119.8
O4—C2—Cd1^iii^	58.55 (13)	C16—C17—H17	119.8
C7—C2—Cd1^iii^	177.30 (18)	C17—C18—C20	116.4 (4)
C8—C3—C4	119.5 (2)	C17—C18—C19	122.6 (3)
C8—C3—C1	120.8 (2)	C20—C18—C19	121.1 (3)
C4—C3—C1	119.7 (2)	C18—C19—H19A	109.5
C5—C4—C3	120.8 (2)	C18—C19—H19B	109.5
C5—C4—H4	119.6	H19A—C19—H19B	109.5
C3—C4—H4	119.6	C18—C19—H19C	109.5
O5—C5—C4	123.8 (2)	H19A—C19—H19C	109.5
O5—C5—C6	116.7 (2)	H19B—C19—H19C	109.5
C4—C5—C6	119.5 (3)	N2—C20—C18	124.2 (3)
C5—C6—C7	120.0 (2)	N2—C20—H20	117.9
C5—C6—H6	120.0	C18—C20—H20	117.9
C7—C6—H6	120.0		
			
O4^i^—Cd1—O1—C1	−70.7 (2)	C3—C4—C5—C6	−1.8 (4)
N1—Cd1—O1—C1	82.4 (2)	O5—C5—C6—C7	−176.5 (3)
N2—Cd1—O1—C1	143.3 (2)	C4—C5—C6—C7	3.6 (4)
O4^ii^—Cd1—O1—C1	−134.8 (2)	C5—C6—C7—C8	−2.4 (4)
O3^i^—Cd1—O1—C1	−5.3 (2)	C5—C6—C7—C2	175.8 (2)
C2^i^—Cd1—O1—C1	−36.7 (2)	O3—C2—C7—C6	−146.8 (3)
O1—Cd1—N1—C9	−43.2 (3)	O4—C2—C7—C6	31.5 (4)
O4^i^—Cd1—N1—C9	102.1 (3)	O3—C2—C7—C8	31.5 (4)
N2—Cd1—N1—C9	−178.2 (3)	O4—C2—C7—C8	−150.3 (3)
O4^ii^—Cd1—N1—C9	−123.8 (3)	C6—C7—C8—C3	−0.5 (4)
O3^i^—Cd1—N1—C9	75.9 (3)	C2—C7—C8—C3	−178.7 (2)
C2^i^—Cd1—N1—C9	86.3 (3)	C4—C3—C8—C7	2.2 (4)
O1—Cd1—N1—C14	129.2 (3)	C1—C3—C8—C7	−179.5 (2)
O4^i^—Cd1—N1—C14	−85.5 (3)	C14—N1—C9—C10	−1.4 (7)
N2—Cd1—N1—C14	−5.9 (2)	Cd1—N1—C9—C10	170.9 (4)
O4^ii^—Cd1—N1—C14	48.5 (3)	N1—C9—C10—C12	0.0 (8)
O3^i^—Cd1—N1—C14	−111.7 (2)	N1—C9—C10—C11'	165.1 (6)
C2^i^—Cd1—N1—C14	−101.4 (2)	N1—C9—C10—C11	−161.0 (7)
O1—Cd1—N2—C20	119.8 (3)	C9—C10—C12—C13	0.7 (8)
O4^i^—Cd1—N2—C20	−32.6 (3)	C11'—C10—C12—C13	−163.1 (7)
N1—Cd1—N2—C20	−172.3 (3)	C11—C10—C12—C13	162.4 (7)
O4^ii^—Cd1—N2—C20	37.6 (3)	C10—C12—C13—C14	−0.2 (7)
O3^i^—Cd1—N2—C20	−88.3 (3)	C9—N1—C14—C13	1.9 (5)
C2^i^—Cd1—N2—C20	−60.2 (3)	Cd1—N1—C14—C13	−170.8 (3)
O1—Cd1—N2—C15	−67.1 (3)	C9—N1—C14—C15	−177.4 (3)
O4^i^—Cd1—N2—C15	140.4 (2)	Cd1—N1—C14—C15	9.9 (4)
N1—Cd1—N2—C15	0.7 (2)	C12—C13—C14—N1	−1.1 (6)
O4^ii^—Cd1—N2—C15	−149.3 (2)	C12—C13—C14—C15	178.1 (4)
O3^i^—Cd1—N2—C15	84.8 (2)	C20—N2—C15—C16	−0.4 (5)
C2^i^—Cd1—N2—C15	112.8 (2)	Cd1—N2—C15—C16	−173.7 (3)
Cd1—O1—C1—O2	14.9 (4)	C20—N2—C15—C14	177.3 (3)
Cd1—O1—C1—C3	−162.18 (18)	Cd1—N2—C15—C14	3.9 (4)
Cd1^iii^—O3—C2—O4	−0.4 (3)	N1—C14—C15—N2	−9.2 (5)
Cd1^iii^—O3—C2—C7	177.8 (2)	C13—C14—C15—N2	171.6 (3)
Cd1^iii^—O4—C2—O3	0.5 (3)	N1—C14—C15—C16	168.4 (4)
Cd1^iv^—O4—C2—O3	−168.2 (3)	C13—C14—C15—C16	−10.9 (6)
Cd1^iii^—O4—C2—C7	−177.7 (2)	N2—C15—C16—C17	1.6 (7)
Cd1^iv^—O4—C2—C7	13.6 (6)	C14—C15—C16—C17	−175.9 (4)
Cd1^iv^—O4—C2—Cd1^iii^	−168.7 (5)	C15—C16—C17—C18	−1.6 (8)
O2—C1—C3—C8	−167.0 (3)	C16—C17—C18—C20	0.5 (7)
O1—C1—C3—C8	10.3 (4)	C16—C17—C18—C19	179.5 (5)
O2—C1—C3—C4	11.3 (4)	C15—N2—C20—C18	−0.8 (6)
O1—C1—C3—C4	−171.5 (3)	Cd1—N2—C20—C18	172.2 (3)
C8—C3—C4—C5	−1.1 (4)	C17—C18—C20—N2	0.7 (6)
C1—C3—C4—C5	−179.3 (3)	C19—C18—C20—N2	−178.3 (4)
C3—C4—C5—O5	178.3 (3)		

Symmetry codes: (i) *x*, −*y*+3/2, *z*−1/2; (ii) −*x*+1, *y*+1/2, −*z*+3/2; (iii) *x*, −*y*+3/2, *z*+1/2; (iv) −*x*+1, *y*−1/2, −*z*+3/2.

### Hydrogen-bond geometry (Å, °)

**Table:**

*D*—H···*A*	*D*—H	H···*A*	*D*···*A*	*D*—H···*A*
O5—H5···O2^v^	0.82	1.86	2.680 (3)	174
C6—H6···O1^iv^	0.93	2.31	3.229 (3)	169
C17—H17···O3^vi^	0.93	2.53	3.355 (5)	147

Symmetry codes: (v) −*x*+1, −*y*+1, −*z*+1; (iv) −*x*+1, *y*−1/2, −*z*+3/2; (vi) −*x*, −*y*+2, −*z*+1.
